# Diagnosis, Characterization and Treatment of Emerging Pathogens

**DOI:** 10.3390/microorganisms11082032

**Published:** 2023-08-08

**Authors:** Shengxi Chen

**Affiliations:** Biodesign Center for Bioenergetics, Arizona State University, Tempe, AZ 85287, USA; shengxi.chen.1@asu.edu; Tel.: +1-480-965-5969

Emerging infectious diseases are perhaps the most rapidly spreading diseases. COVID-19 is an infectious disease caused by severe acute respiratory syndrome coronavirus 2 (SARS-CoV-2). The outbreak of COVID-19 started in December 2019 and has infected billions of people, causing about 7 million deaths worldwide to date [1]. Multiple SARS-CoV-2 variants emerged globally, with some demonstrating increased transmissibility or potential resistance to antibodies. Variants of concern, such as the Alpha, Beta, Gamma, Delta, and Omicron variants, caused more fatal syndromes [2]. To date, the world is still suffering from this virus. Another example is Dengue, whose clinical syndrome caused by the dengue virus, presents with a range of manifestations ranging from self-limited febrile illness to hemorrhagic fever and shock, resulting in death [3]. The global incidence of dengue virus infections has increased 30-fold during the last 50 years, with an estimated 50–100 million symptomatic infections occurring each year and over half of the world’s population living at risk of infection [3]. At the same time, there are many other different emerging pathogens, such as malaria, monkeypox, Zika, Ebola, West Nile, and diarrheagenic E. coli, threatening the health of billions of people worldwide.

A comprehensive understanding of the structures of these emerging pathogens and their interactions with hosts is important to develop effective therapeutic and preventive approaches. SARS-CoV-2 is formed by a single-strand positive-sense RNA (~29.9 kb), 16 non-structural proteins (nsp1—16), and four structural proteins—nucleocapsid protein (N), envelope protein (E), membrane protein (M), and spike protein (S) [4]. The nucleocapsid protein forms the capsid outside the SARS-CoV-2 RNA genome, and the RNA genome is further packed by envelope proteins, membrane proteins, and spike proteins. The non-structural proteins mediate SARS-CoV-2 RNA transcription, protein translation, protein splicing, and viral replication. Among them, the proteins nsp7, nsp8, and nsp12 form the RNA-dependent RNA polymerase (RdRp) to transcript viral RNA [4]. SARS-CoV-2 is an airborne-transmission virus. The trimers of the S protein of SARS-CoV-2 bind to the angiotensin converting-enzyme 2 (ACE2) receptor of host cells to initiate the infection [5]. The S protein undergoes proteolytic cleavage by several host proteases, such as cathepsin L, furin, and TMPRSS2, resulting in virus–host membrane fusion. The viral RNA is released into the host cytoplasm to replicate its genetic material and assemble new viral particles utilizing the host and its own machinery.

The early and accurate diagnosis of COVID-19 allows for appropriate treatment and timely isolation of infected individuals, reducing the risk of further transmission. The diagnosis of COVID-19 typically involves a combination of clinical syndromes, diagnostic imaging (e.g., chest X-rays or computed tomography scans), and laboratory testing [6]. The primary laboratory methods for detecting COVID-19 are through the detection of the RNA and protein antigens (N, S proteins) of the SARS-CoV-2 virus [7]. The detection of human antibodies (IgA, IgG, IgM) is used to evaluate the infection history of the SARS-CoV-2 virus. Reverse transcription polymerase chain reaction (RT-PCR) tests are the gold standard for detecting the presence of the SARS-CoV-2 virus. These RT-PCR tests detect the specific sequences of SARS-CoV-2 RNA in respiratory samples, such as nasopharyngeal swabs or throat swabs. The process involves amplifying and analyzing the conserved viral RNA sequences to confirm the presence of the SARS-CoV-2 virus. The RT-PCR tests are highly accurate but require specialized laboratory equipment and may take several hours to produce results [8]. Isothermal amplification techniques have been developed to detect SARS-CoV-2 RNAs as alternative methods to RT-PCR. Reverse transcription loop-mediated isothermal amplification (RT-LAMP), nucleic acid sequence-based amplification (NASBA), rolling circle amplification (RCA), recombinase polymerase amplification (RPA), and transcription-mediated amplification (TMA) are used for isothermal-based SARS-CoV-2 RNA tests. These isothermal detection methods are coupled to a variety of portable devices for readouts, making them more accessible and user-friendly. Next-generation sequencing (NGS) technology is capable of comprehensively sequencing the whole SARS-CoV-2 genome and identifying newly emerging variants, and this technology has been used to discover SARS-CoV-2 and its variants. All the RNA diagnosis methods mentioned above require high-cost materials and highly skilled bioinformatics staff, thus restricting their broad use. Comparatively, rapid antigen/antibody tests are designed to detect specific viral proteins or human antibodies associated with the SARS-CoV-2 virus. These tests provide relatively faster results (usually within 15–30 min) and are less expensive compared to RT-PCR tests [8]. In addition, the rapid antigen tests are suitable to be used at home by any individual. Lab diagnosis of the SARS-CoV-2 virus is crucial for providing appropriate treatments and interventions to infected individuals, as well as tracking the spread of COVID-19 on a local, national, and global scale. It is also useful to assess the prevalence of the virus, identify hotspots, and control the spread of the virus. However, there are several unmet clinical needs for the diagnosis of COVID-19. RT-PCR and its alternative methods for RNA detection require several hours to produce results, thus delaying the decisions for timely treatment and suitable isolation of SARS-CoV-2-infected patients. It is an urgent need to develop a faster RNA test that could provide results within minutes, allowing for more immediate decisions in the future. Antigen/antibody tests are more rapid alternatives to PCR tests, but they are less sensitive and may have a higher rate of false-negative results, especially in asymptomatic individuals or those with low viral loads. Thus, improvements in the sensitivity and specificity of antigen/antibody tests in the future are needed to enhance their utility in diagnosing COVID-19.

Efforts to control the SARS-CoV-2 virus include a combination of vaccination campaigns, viral testing, contact tracing, mask wearing, social distancing, and adapting public health measures as new information emerges. However, these efforts cannot thoroughly prevent the spread of SARS-CoV-2 infection. Numerous patients still require drug treatment for SARS-CoV-2 infection. Remdesivir is an antiviral drug that was initially developed to treat Ebola. It targets the RNA-dependent RNA polymerase (RdRp) of the SARS-CoV-2 virus to inhibit viral replication [9]. It has been approved in several countries for the treatment of COVID-19 in certain patient populations, aged 12 and older. The FDA has authorized molnupiravir for emergency use as a drug to treat COVID-19 patients, which is another RdRp inhibitor. Molnupiravir is used by RdRp to transcript viral RNA, thus increasing the frequency of viral RNA mutations and impairing SARS-CoV-2 replication in humans [10]. Another anti-SARS-CoV-2 drug, Paxlovid (a combination of two drugs—nirmatrelvir and ritonavir) has also been authorized for emergency use by the FDA. Nirmatrelvir is a peptidomimetic inhibitor of SARS-CoV-2’s main protease (Mpro). It covalently binds to the catalytic cysteine (Cys145) residue of Mpro and prevents the virus from processing the polyprotein precursors that are required for viral replication [11]. Ritonavir is an HIV protease inhibitor, which inhibits the metabolizing enzyme cytochrome P450 3A (CYP3A) to lengthen the half-life of nirmatrelvir. In Paxlovid, rintonavir acts as a pharmacological enhancer for nirmatrelvir [12]. To date, five anti-SARS-CoV-2 mAb products (bebtelovimab, sotrovimab, bamlanivimab + etesevimab, casirivimab + imdevimab, and tixagevimab + cilgavimab) have received Emergency Use Authorizations (EUA) from the FDA to treat outpatients with mild to moderate COVID-19 [13]. These antibodies bind to nonoverlapping epitopes of the S protein of SARS-CoV-2. Nevertheless, they are not currently authorized for use because the dominant Omicron subvariants are not susceptible to these products. COVID-19 convalescent plasma has also received EUA from the FDA for the treatment of hospitalized patients with COVID-19. In addition, three immunosuppressive drugs, baricitinib, dexamethasone, and tocilizumab, have been used in some cases of severe COVID-19 to control the excessive immune response [14]. To date, ongoing research and clinical trials continue to explore new drugs and therapeutic strategies for COVID-19.

Dengue virus is a member of the family of flaviviruses and is related to yellow fever, West Nile, Zika, and Japanese encephalitis. It is a rapidly spreading endemic viral disease in subtropical and tropical regions of the world [3]. Dengue virus is a mosquito-borne pathogen frequently transmitted by *Aedes aegypti*, *Aedes albopictus*, and *Aedes polynesiensis*. Currently, this disease threatens more than 2.5 billion people in more than 100 countries including the Americas, the Western Pacific, Southeast Asia, the Eastern Mediterranean, and Africa. It is estimated that 50–100 million dengue infections cause 24,000 deaths annually.

Dengue virus infection can cause severe systemic diseases, such as high-grade fever, bleeding, and shock, which results in a fatality rate as high as 10% without proper treatment in one week [3]. However, the early recognition of dengue and proper treatment reduce the fatality rate to <1%. To date, no vaccine or specific drug against dengue virus has been successfully developed. Thus, the early detection of dengue infection is extremely important to cure patients and prevent the spread of the epidemic. Dengue virus is a single-stranded encapsulated RNA virus containing a positive-sense RNA of 11 kb in length, multiple copies of three structural proteins (the capsid protein (C), membrane protein (M), and the envelope protein (E)), and seven nonstructural proteins (NS1, NS2A, NS2B, NS3, NS4A, NS4B, and NS5) [15]. Infection with dengue virus causes a broad spectrum of clinical symptoms, many of which are similar to other infections such as flu virus. Thus, a diagnosis of dengue virus infection based only on clinical syndromes is not reliable. An early laboratory diagnosis is required to confirm the dengue virus infection since more than 50% of patients have a mild non-specific fever or are asymptomatic at the early stage of the disease. During 1–5 days after the onset of symptoms, the dengue virus can be isolated from patients to diagnose the infection and the serotypes. However, this technique requires tissue culture assays, which require 1–2 weeks for detection. Another effective diagnostic method is to detect antibodies and/or antigens of dengue in blood samples. Recently, rapid diagnostic tests (RDT) that simultaneously detect the NS1 antigen and IgM antibody of the dengue virus have been reported. Unfortunately, the cross-reactivity between flaviviruses limits the sensitivity and specificity of these RDT methods [16,17]. To date, the diagnosis of dengue viral RNA in patient’s blood, serum, or plasma using a reverse transcriptase PCR (RT-PCR) and real-time RT-PCR (rRT-PCR) have been shown to have the highest sensitivity and specificity of all early-detection methods [18,19]. RT-PCR and rRT-PCR methods require multiple thermocycling steps, which is time-consuming and requires special thermocycling equipment. To avoid these multiple thermocycling steps, isothermal amplification methods (RT-LAMP, RT-RPA, and NASBA) that require a single-step isothermal RNA amplification temperature have been used to detect four dengue virus serotypes [20]. Even though these isothermal amplification methods require only a single thermocycling step (a single melting step at 60–65 °C and an amplification reaction at 37–42 °C), they still require special temperature control equipment. Recently, tandem toehold-mediated displacement reactions (tTMDR) were developed to amplify the dengue RNA signal at room temperature without any additional equipment [21,22]. This method has been used to detect four serotypes of dengue virus with a limit of as low as six copies of RNA per sample. However, these RNA molecular assays at the point of care have been hindered by expensive reagents and equipment, high test complexity, and the extensive laboratory infrastructure required for performance. In addition, dengue is prevalent in many low-resource settings, where access to sophisticated diagnostic tools may be limited. Therefore, it is required to develop rapid, accurate, and economical molecular tests that improve clinical care and inform targeted public health interventions in the future, especially in remote or underserved areas.

Currently, there is no specific antiviral drug available to treat dengue fever. Treatment for dengue mainly focuses on supportive care to manage symptoms and prevent complications. Research is ongoing to develop specific antiviral drugs for dengue. Several potential inhibitors targeting NS3, NS5, and RdRp of the dengue virus are being investigated [23]. However, no specific antiviral drug had been approved for the treatment of dengue to date. In addition, there is no approved specific antiviral drug to treat other flaviviruses either. More efforts are required to develop effective drugs to inhibit these flaviviruses in the future.

In summary, emerging infectious diseases have caused serious damage to human health and continue to threaten the health of billions of people worldwide. Research on the characterization of specific genes or protein targets for the detection or treatment of emerging pathogens, novel methods and strategies for early detection of emerging pathogens, and novel agents to inhibit emerging pathogens are required to fight against these emerging infectious diseases.
